# Supplementary material for: Transcriptomic Changes of Murine Visceral Fat Exposed to Intermittent Hypoxia at Single Cell Resolution
Source: Int J Mol Sci. 2020 Dec 29;22(1):261. doi: 10.3390/ijms22010261 (PMC7795619; doi:10.3390/ijms22010261)
Supplement: Supplementary file 1 [file ijms-22-00261-s001.zip › Supplementary figures.pdf]

Supplementary Figures

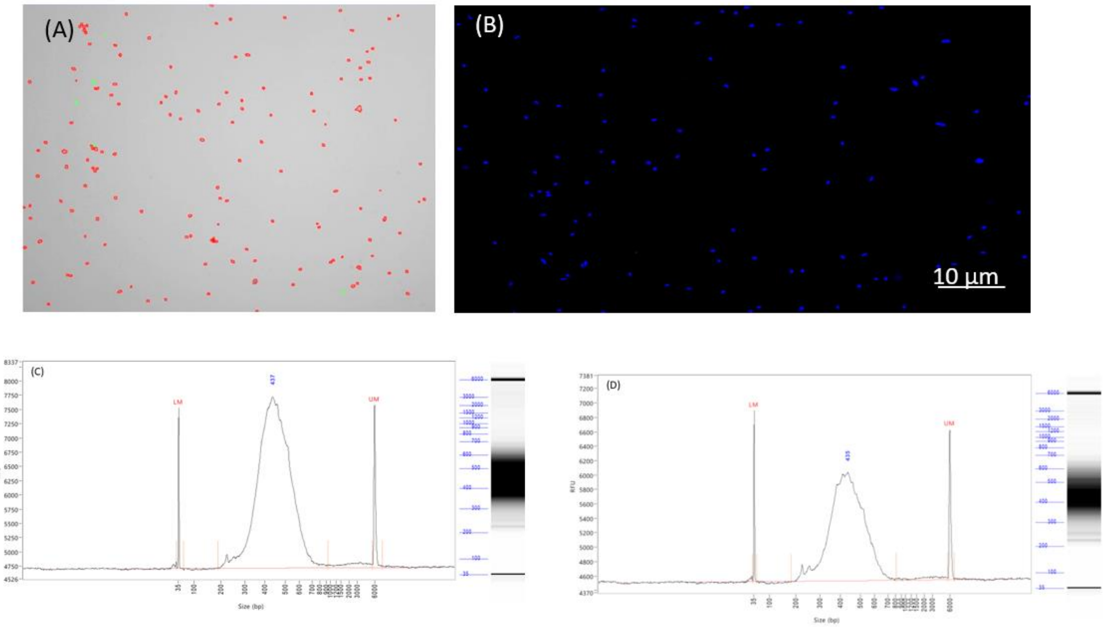

Figure S1. Quality control and characteristics of single nuclei RNA-seq (snRNA-seq) in eWAT. (A) Single nuclei were determined using Countess® II Automated Cell Counter. (B) Fluorescent microscopic analysis of stained single nuclei with DAPI. (C) Fragment Analyzer for single nuclei of cDNA library from RA condition. (D) Fragment Analyzer for single nuclei of cDNA library from IH.

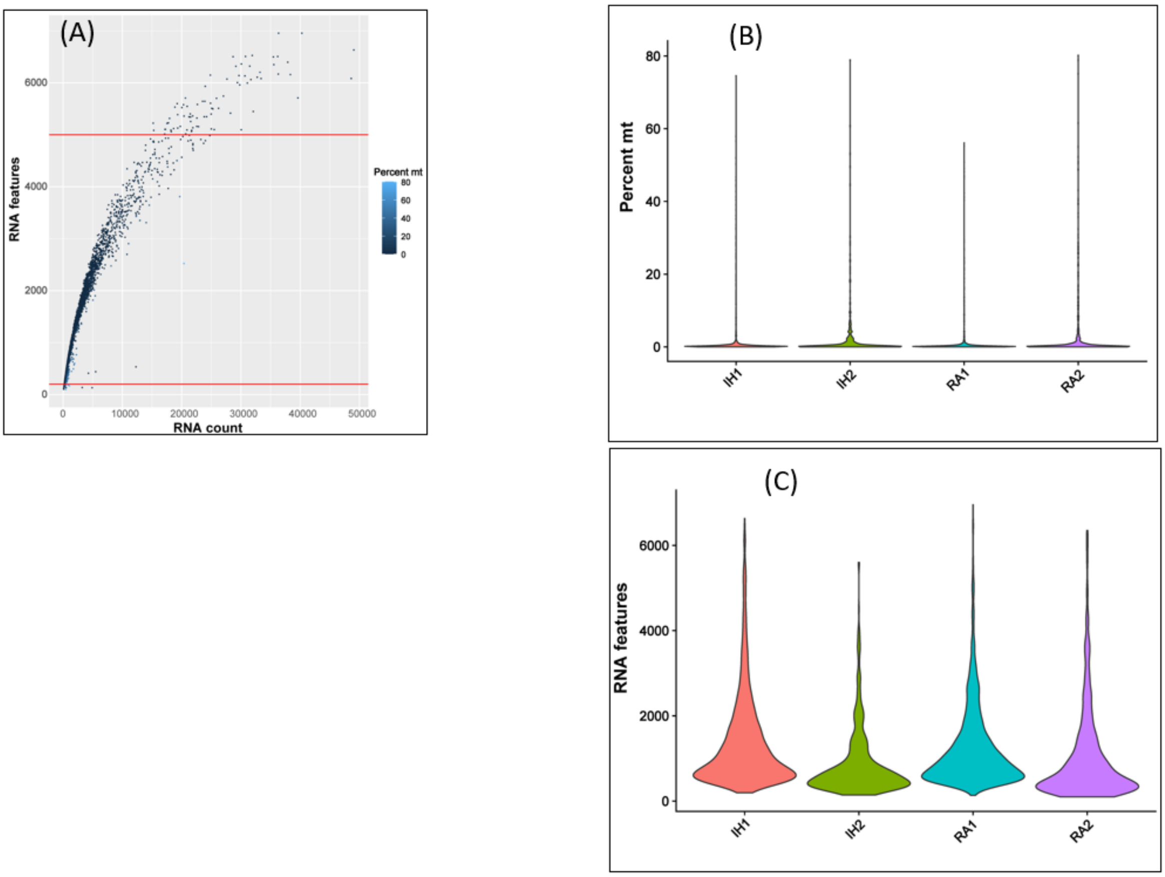

Figure S2. Quality controls for single nuclei cells using Feature and Violin plots. (A) Feature_plot shows the number of gene counts (Y axis) and the number of RNA reads (X axis). The flat "bottom" of each "violin" corresponds to a minimum cutoff of 200. Each cell is colored by the percentage gene counts of attributed to mitochondrial genes. (B) Violin plot represents percent.mt percentage (%) of measured gene expression in each cell that is attributed to mitochondrial genes. (C) Violin plot represents nCount_RNA number of reads mapped to RNA gene features. These analyses may impose a maximum for this number, to try to avoid including data from "doublets " (i.e. two cells accidentally captured together, which makes it practically impossible to distinguish reads from one cell or the other.

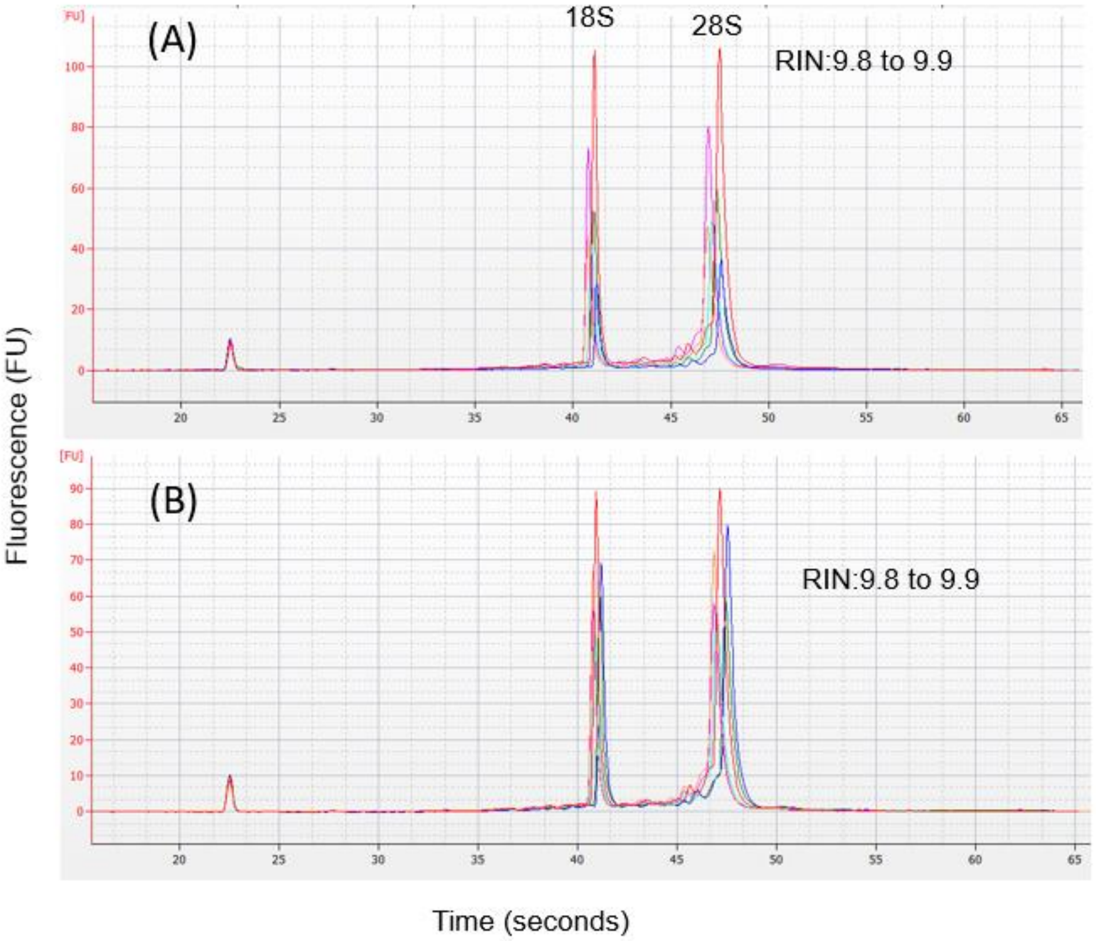

Figure S3. Quality control assessment of total RNA used for RNA-seq and qRT-PCR analyses. Total RNAs were isolated from eWAT of mice exposed to IH for 6wks and matched control (RA). Total RNA samples were resolved on capillary electrophoresis gels, and a representative electropherogram from RA samples (A), and IH samples are shown (B). The fluorescence is depicted on the Y-axis and the time (s) on the X-axis.

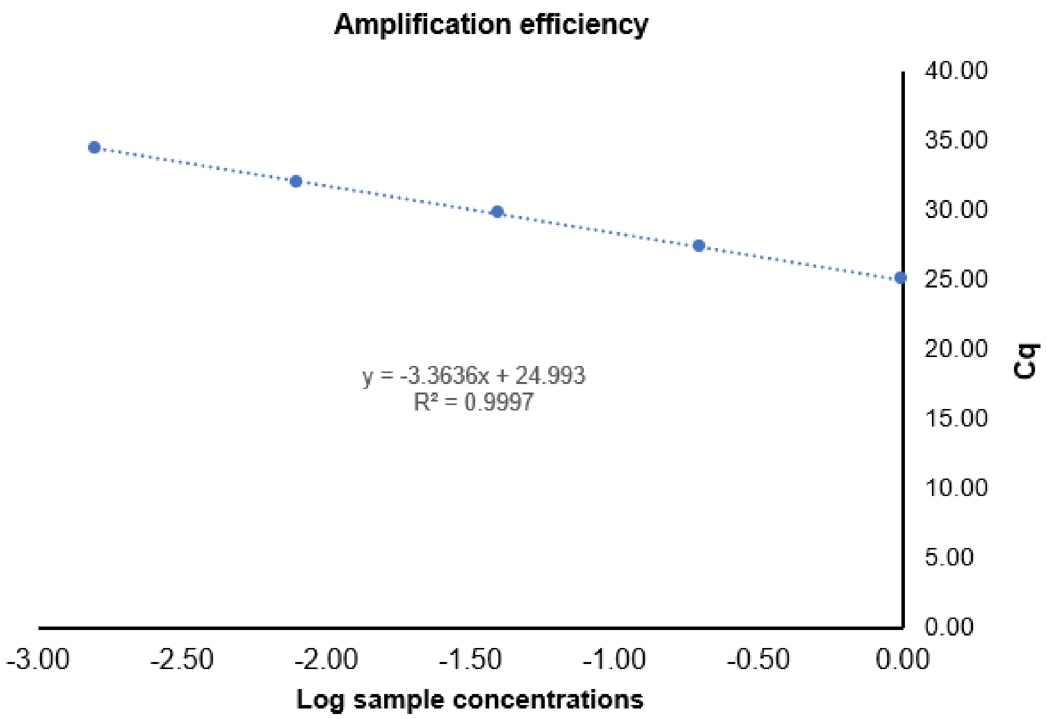

Figure S4. Amplification efficiency for quality control of RNA inhibition. cDNAs were prepared as described in Methods section, and the samples were diluted to be 1, 0.2, 0.04, 0.008, and 0.0016 using b-actin gene. The primer efficiency was calculated for primer efficiency = (10^(-1/The Slope Value)-1)*100 Slope= -3.3636, primer efficiency= 98%. Indicating no RNA inhibition.
